# Lack of association between pretreatment glutamate/GABA and major depressive disorder treatment response

**DOI:** 10.1038/s41398-025-03292-9

**Published:** 2025-03-02

**Authors:** Feiyang Dai, Kenneth Wengler, Xiang He, Junying Wang, Jie Yang, Ramin V. Parsey, Christine DeLorenzo

**Affiliations:** 1 UC Berkeley, Berkeley, CA USA; 2 Department of Psychiatry, Icahn School of Medicine at Mount Sinai, New York, NY USA; 3 Department of Radiology, Northwell Health, New York, NY USA; 4 Department of Applied Mathematics and Statistics, Stony Brook University, Stony Brook, NY USA; 5 Department of Family, Population, and Preventive Medicine, Stony Brook University, Stony Brook, NY USA; 6 Department of Psychiatry, Stony Brook University, Stony Brook, NY USA; 7 Department of Biomedical Engineering, Stony Brook University, Stony Brook, NY USA; 8 Department of Psychiatry, Columbia University, New York, NY USA

**Keywords:** Predictive markers, Molecular neuroscience

## Abstract

Studies have shown gamma-amino-butyric acid (GABA) and Glx (a combination of glutamate and glutamine) to be altered in major depressive disorder (MDD). Using proton Magnetic Resonance Spectroscopy (^1^H-MRS), this study aimed to determine whether lower pretreatment GABA and Glx levels in the medial frontal cortex, a region implicated in MDD pathophysiology, are associated with better antidepressant treatment response. Participants with MDD (*N* = 74) were antidepressant naïve or medication-free for at least three weeks before imaging. Two MEGA-PRESS ^1^H-MRS acquisitions were collected, interleaved with a water unsuppressed reference scan. GABA and Glx concentrations were quantified from an average difference spectrum, with preprocessing using Gannet and spectral fitting using TARQUIN. Following imaging, participants were randomized to escitalopram or placebo for 8 weeks in a double-blind design. Multivariable logistic regression models were applied with treatment type and age as covariates. Bayes Factor hypothesis testing was used to interpret the strength of the evidence. No significant association was found between pretreatment Glx, GABA, or Glx/GABA and depression remission status or the continuous outcome, percent change in symptom severity. In an exploratory analysis, no significant correlation was found between pretreatment Glx, GABA or Glx/GABA and days to response. Bayes factor analysis showed strong evidence towards the null hypotheses in all cases. To date, there are no replicated biomarkers in psychiatry. To address this, well-powered, placebo-controlled trials need to be undertaken and reported. The present analysis suggests pretreatment GABA, Glx, or their ratio cannot predict antidepressant treatment response. Future direction including examining glutamate and glutamine separately or examining biological subtypes of MDD separately.

Trial Name: Advancing Personalized Antidepressant Treatment Using PET/MRI.

Registration Number: NCT02623205 URL: https://clinicaltrials.gov/ct2/show/NCT02623205

## Introduction

Major depressive disorder (MDD) affects over 300 million people worldwide and is the leading cause of global disability [1]. Current treatment guidelines recommend a “trial-and-error” paradigm [2]; however, successful treatment using this paradigm can take a year or more [3]. Biomarkers that could predict treatment success prospectively, or early in the course of treatment, would allow for a personalized medicine approach, reducing time spent on ineffective treatment, shortening patients’ suffering, and reducing the overall cost burden of MDD [4].

Since 90–99% of brain neurons are either glutamatergic (excitatory) or GABAergic (inhibitory) [5], the physiological balance of excitatory/inhibitory signals may be crucial to the proper functioning of the Central Nervous System (CNS) [6]. In support of this hypothesis, disruption of glutamate and GABA levels in MDD has been reported by multiple studies [7–9]. Relatedly, antidepressant treatments influence these neurotransmitters; for example, the rapid antidepressant ketamine, an NMDA antagonist [10–12], and d-cycloserine, an antibiotic that works as a positive modulator of NMDA receptors [13, 14]. Modulation of glutamate and GABA has also been implicated in multiple antidepressant treatments without glutamate/GABA as primary targets, including Cognitive Behavioral Therapy (CBT) [15–18], Deep Brain Stimulation (DBS) [19–22], Electroconvulsive Therapy (ECT) [23–28], repetitive Transcranial Magnetic Stimulation (rTMS) and Selective Serotonin Reuptake Inhibitors (SSRIs) [29].

If modulation of glutamate and GABA is required for treatment response, then pretreatment levels of these metabolites may provide insight into this eventual response. However, as summarized in Table 1, few previous studies have used Proton Magnetic Resonance Spectroscopy (^1^H-MRS) to non-invasively examine glutamate and GABA levels as potential treatment predictors and, when performed, this treatment prediction has yielded mixed results. This is likely due to the small sample sizes (only one study had >30 MDD participants), variability in regions examined [30], and variable inclusion of those with treatment-resistant depression (TRD), defined as not responding to at least two previous antidepressant treatment trials [31]. Also, to our knowledge, no studies examined whether pretreatment glutamate or GABA levels were associated with response to placebo. This would provide critical insight into the specificity of the predictive signal.

**Table 1:** Summary of previous studies using pretreatment magnetic resonance spectroscopy (^1^H-MRS) to predict efficacy of antidepressant treatment in MDD, listed in chronological order.

Author	Subjects	Males: Females	Treatment Resistant Depression	Examined Regions	Treatment used	Pretreatment Level in MDD	Change with treatment	Prediction
Sanacora, G., et al. [29]	11 MDD	7:4	Not included	OCC	SSRI		GABA increased after SSRI treatment (GABA)	No correlation between GABA and response (however, lowest GABA level was associated with largest symptom improvement) (GABA)
Luborzewski, A., et al. [11]	17 MDD	2:15	Included	Left ACC, Left DLPFC	rTMS		Glu in the DLPFC increased in responders but decreased in non-responders (Glu)	Lower Glu in the DLPFC predicted response to rTMS (Glu)
Block, W., et al. [94]	13 MDD 10 HC	8:5 6:4	Included	Hippocampus	citalopram or nortriptyline	Reduced Glx/Cr and Gln/Glx ratio in MDD (Glx/Cr, Gln/Glx)		Lower NAA and Cho correlated with response (NAA, Cho)
Merkl, A., et al. [23]	25 MDD 27 HC	7: 18	Only	ACC	ECT	Lower Glu in MDD (Glu, NAA, tCR)		Higher Glu predicted response (Glu, NAA, tCR)
Salvadore, G., et al. [10]	14 MDD	9: 5	Included	PFC	Ketamine		Glx/Glu increased after ketamine (Glx, Glu, GABA)	Lower Glx/Glu predicted response (Glx, Glu, GABA)
Abdallah, C. G., et al. [15]	30 MDD	10: 20	Not Included	OCC	CBT		Glu decreased in responders (Glu)	Higher Gln predicted response (GABA, Glu, Gln)
Brennan, B.P., et al. [71]	19 unmedicated MDD 10 HC	16: 3	Not Included	pgACC	SSRI	Lower Glu and GABA in MDD (Glu, GABA)	GABA increased with response (Glu, GABA)	Lower GABA predicted response (Glu, GABA)
Njau, S. et al. [24]	50 MDD patients 33 HC	23:27 14:19	Only	ACC Left Hipp	ECT	Lower Glx in MDD (Glx)	Decreased Glx in Left Hipp and increased Glx in sgACC associated with response (Glx)	Lower Glx levels predicted response (Glx)
Evans, J.W., et al. [95]	20 MDD patients 17 HC	8:12 5:12	Only	pg ACC	Ketamine	No significant differences (Glu, Gln)	Increased Glu after infusion. (Glu)	No association. (Glu)
Clark, D.L., et al. [76]	16 patients with TRD	7:9	Only	rACC	DBS			Lower Glu predicted response (Glu)
Bhattacharyya. P. et al. [96]	7 MDD patients	4:3	Only	Left DLPFC	rTMS		Change in Glx/tCr correlated with response (GABA/tCr, Glx/ tCr)	Higher Glx/tCr predicted response (Glx/tCr, GABA/tCr)
Dong Z. C. et al. [97]	8 bipolar depression patients	1:7		vmPFC, ACC	D- cycloserine			Lower Glu after treatment associated with response. (Glu)
Ermis, et al. [98]	30 patients with unipolar MDD	10:20	Not Included	ACC	ECT		Elevated Glx levels associated with response (Glx)	Higher Glx predicted remission (Glx)
Godfrey, K.E.M., et al. [75]	27 MDD	16:11	Only	DLPFC, M1	rTMS		Glx levels increased with response (Glx)	Lower pretreatment Glx associated with response (Glx)
Gonsalves et al. [99]	27 MDD	13:14	Only	dACC	rTMS			Lower baseline Glu and Glx predicted greater response (Glu, Glx)

Parentheses in the last two columns indicate the brain metabolites examined in the study.

*ACC* anterior cingulate cortex, *CBT* cognitive behavioral therapy, *Cho* choline, *dACC* dorsal anterior cingulate cortex, *DBS* deep brain stimulation, *DLPFC* dorsolateral prefrontal cortex, *ECT* electroconvulsive therapy, *GABA* gamma-aminobutyric acid, *Gln* glutamine, *Glu* glutamate, *Glx* glutamate and glutamine, *GSH* glutathione, *HC* healthy controls, *HDRS* hamilton depression rating scale, *Hipp* hippocampus, *MDD* major depressive disorder, *mPFC* medial prefrontal cortex, *M1* primary motor cortex, *NAA* n-acetyl aspartate, *OCC* occipital cortical cortex, *PCC* posterior cingulate cortex, *pgACC* pregenual anterior cingulate cortex, *PFC* prefrontal cortex, *rACC* rostral anterior cingulate cortex, *rTMS* repetitive transcranial magnetic stimulation, *SSRI* selective serotonin reuptake inhibitor, *tCR* total creatine, *vmPFC* ventromedial prefrontal cortex.

To overcome these challenges, this study involved a large cohort (>70 participants, 21 TRD) of participants with MDD, with imaging performed prior to a double-blind placebo-controlled trial of escitalopram. ^1^H-MRS was performed in the medial frontal cortex, encompassing part of the dorsal anterior cingulate cortex (dACC) and parts of the frontal pole, as in [32]. While the medial frontal cortex, including the medial prefrontal cortex and the ACC [33, 34], has been consistently found to contribute to the chronic stress response in MDD [34–37], the ACC, specifically, serves as an important “hub” of the default mode network (DMN), and its overactivation can result in increased negative self-referential rumination, which is associated with more severe depressive symptoms [38]. Lower Glx, a joint measure of glutamine (Gln) and glutamate (Glu) that is commonly used in ^1^H-MRS studies, and GABA levels have been observed in the ACC and frontal cortices of MDD patients (see Table 1).

Based on the above, our main hypotheses are that: (1) participants with MDD who exhibit lower pretreatment Glx and GABA levels are more likely to remit (defined as a 50% or more reduction in the Hamilton Depression Rating Scale (HAM_17_) from pretreatment values and final HAM_17_ < 7 [39]) (8 out of 15 studies examining treatment response prediction in MDD (Table 1) implicated lower pretreatment GABA or Glx level as predictive markers); and (2) pretreatment Glx and GABA levels of participants with MDD will inversely correlate with the magnitude of treatment response.

We hypothesize that the same neural signature will be predictive of both SSRI and placebo response though the possibility of differential prediction will be examined. This is based on multiple clinical trials that have reported similar response rates to placebo and SSRIs [40–42]. Moreover, studies comparing the neurobiological underpinnings of placebo response to various antidepressant treatment modalities, including CBT, DBS, rTMS and SSRI, have found common treatment-induced brain changes in prefrontal, posterior, and parietal cingulate and subgenual cingulate with placebo and SSRI treatment, that differ from the other treatments. Therefore, common biological mechanisms between SSRI and placebo have been hypothesized [43–46]. Further, multiple studies have shown that patients with MDD who respond to placebo often exert greater response to subsequent SSRI treatment [47–49], partially validating a shared mechanism of response.

Further, in this study, depression severity was assessed throughout the treatment course. Therefore, in an exploratory analysis, the relationship between pretreatment Glx and GABA and days to antidepressant response (defined as a 50% or more reduction in the HAM_17_ from pretreatment values) was explored. Conventional antidepressants generally take 4–6 weeks to exert a beneficial effect [50]. However, early (within 1 week) SSRI responses have been reported in some patients [51, 52]. Early improvement in response to antidepressants has been associated with greater chance of achieving remission at the end of 8 week. Thus, there are similarities between prediction of 8-week remission and days to response. Most of the studies examining response time have used only clinical characteristics as predictors [53]. A neurobiological measure could supplement or replace such assessments.

Recent evidence suggests that glutamate or GABA measures may be related to this treatment response time. Ketamine, as an example, can produce an antidepressant response within hours through a transient burst of glutamate caused by the disinhibition of GABAergic interneurons in the brain [54, 55], implying that the quick onset of antidepressant action may be closely related to GABAergic and glutamatergic action. It was, therefore, hypothesized that those who exhibit lower GABA and Glx levels prior to treatment will show an earlier onset of response, since the change in GABA and Glx may be greater in those who have lower pretreatment values.

## Methods

### Participants

**Fig. 1:**
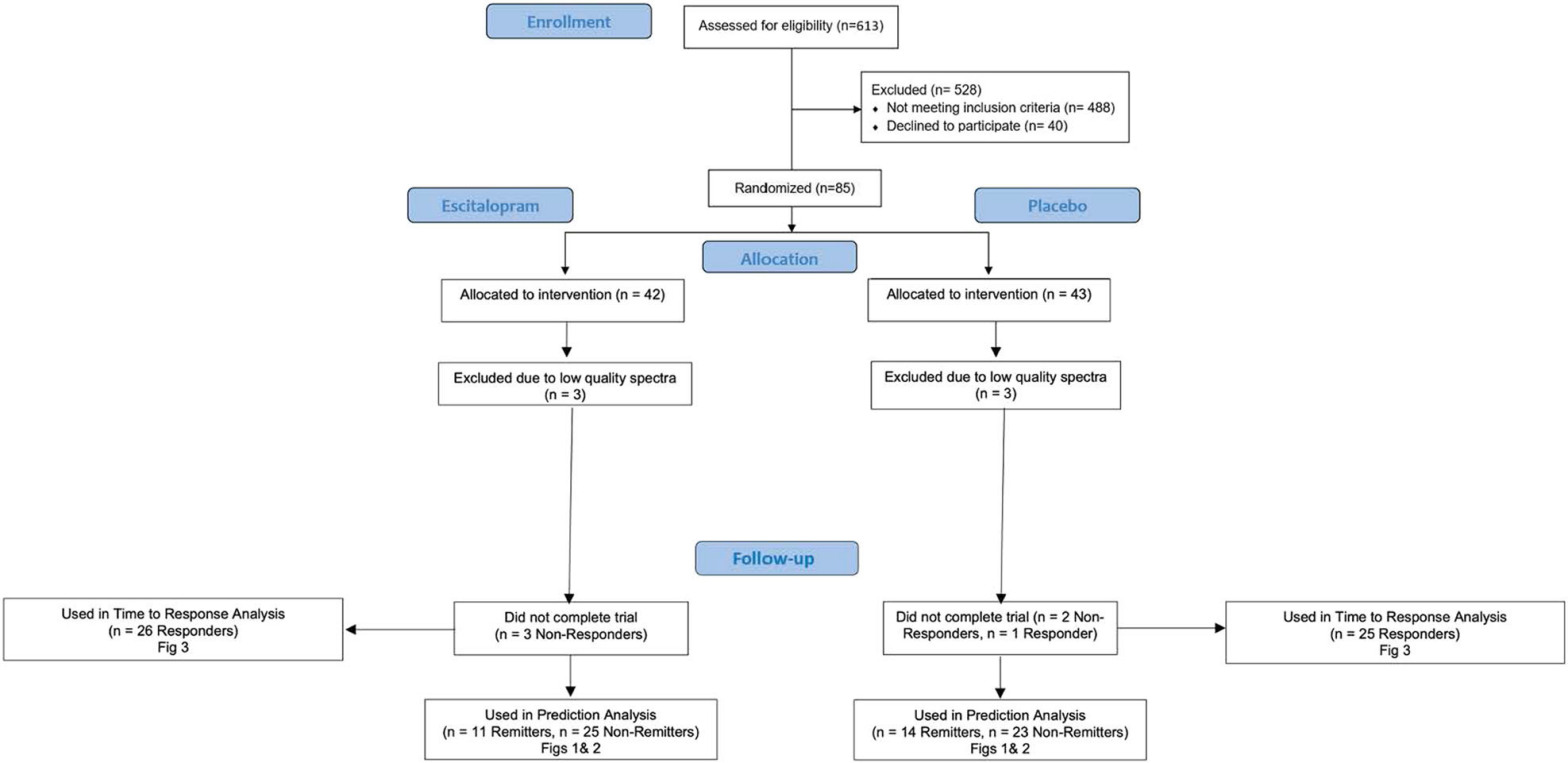
CONSORT Diagram.

Study approval was obtained from Stony Brook University’s Institutional Review Board. Participants (*N* = 85), meeting the DSM-IV criteria for current MDD, were recruited by advertising from the local area and received at least one imaging session (see CONSORT diagram, Fig. 1). All participants signed informed consent after receiving a complete description of the study and were at least 18 years old. Participants met the DSM-IV criteria for a major depressive episode verified by trained rater with the SCID-IV [56, 57] and a score of 22 or higher on the Montgomery-Asberg Depression Rating Scale (MADRS [58], which is considered moderate depression in multiple clinical trials [59]. The MADRS was used for inclusion/exclusion assessment while the HAM_17_ was used to evaluate depression improvement to avoid any inflation of the MADRS that may occur at screening from affecting assessment of remission status. Exclusion criteria were evaluated through clinical judgement of the clinician and the study team and included: current efficacious antidepressant treatment, contraindications to escitalopram including previous failure of escitalopram therapy, ECT within 6 months, lifetime history of psychosis or bipolar disorder, actively suicidal, high potential for excessive substance use during the study period, significant active physical illness, significant neurological deficits, or contradictions to MRI or PET imaging (e.g., metal implants, current pregnancy, or breastfeeding). (PET was part of the exclusion criteria because imaging was performed on a simultaneous PET/MRI [60], see below).

### Treatment

All participants were screened for eligibility by the study clinicians and trained psychological raters. Participants were either medication naïve or medication free for 3 weeks after completing ineffective psychotropic medication washout before study initiation. (Those on effective treatment were excluded from the study.) Washout was completed over a maximum of six weeks before the 3-week psychotropic medication free period. Supportive clinical management was provided during this time. Participants were randomized through a parallel, double-blind design to treatment with either placebo or escitalopram after pretreatment imaging.

Group allocation for all participants was determined at study initiation by pseudo-random allocation scheme (1:1 ratio) generated by the study pharmacist with the software Research Randomizer (http://www.randomizer.org/). Participants met with the study clinician in person approximately each week for the first four weeks, and every other week for the following month. The maximum dosage of medication was 30 mg (3 pills of SSRI or placebo), with a ramp-up period of dosage in intervals of 10 mg of escitalopram in week 1, 20 mg in week 2 and 3, and 30 mg in weeks 4–8. This schedule was altered in the event of treatment intolerance. All participants in the escitalopram cohort remaining in the study at 8 weeks tolerated a dose of 30 mg.

The HAM_17_ was administered prior to pretreatment imaging and after ~8 weeks of treatment, prior to unblinding. The percent decrease between pretreatment and posttreatment HAM_17_ (depression severity decrease) was used as the primary outcome measure. After the study, all participants were offered open treatment.

### Imaging acquisition

**Fig. 2:**
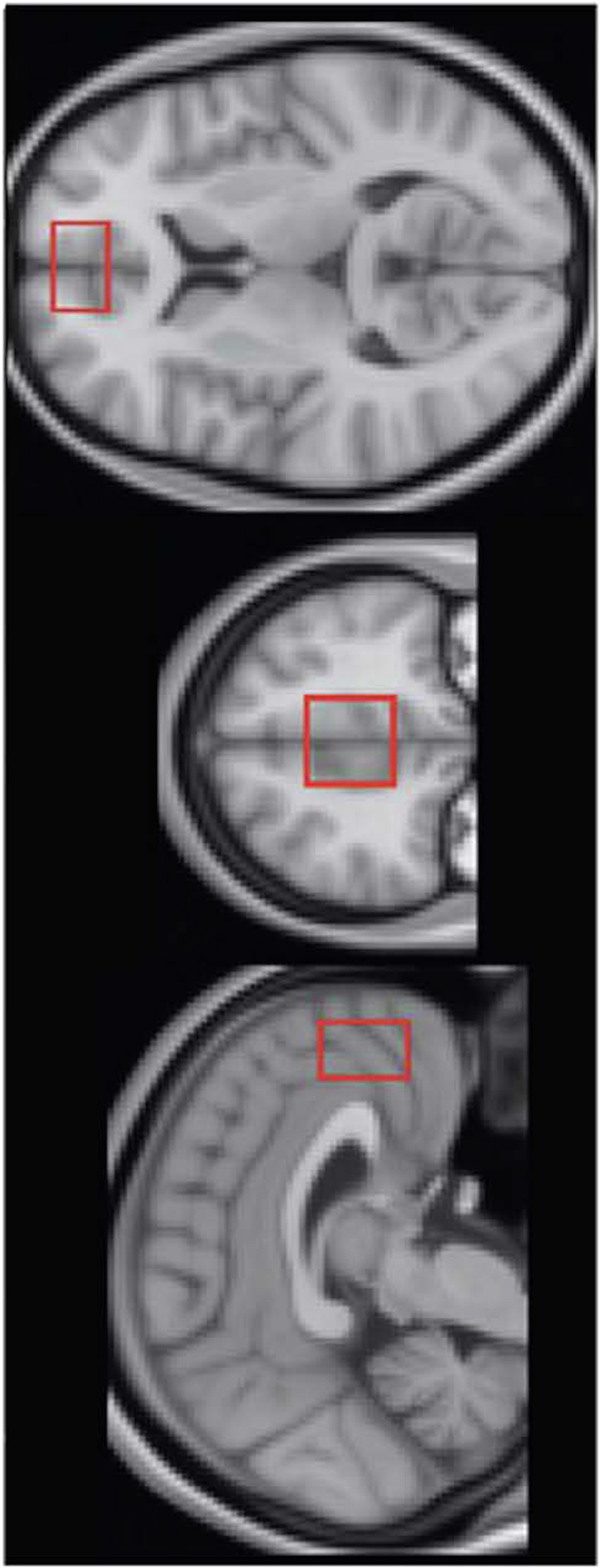
Sample 1H-MRS Location. ^1^H-MRS voxel size and location in the medial frontal cortex, encompassing part of the dorsal anterior cingulate cortex and parts of the frontal pole, shown in the sagittal (left), coronal (middle), and axial (right) views.

All participants in this study were part of a larger study using simultaneous Positron Emission Tomography (PET) and MRI. MRI was performed on a 3 T Siemens mMR scanner with a 12-channel head coil (Siemens, Erlangen, Germany). Anatomical T1-weighted 3D magnetization prepared rapid gradient echo (MPRAGE) images were acquired with the following parameters: repetition time/echo time/inversion time (TR/TE/TI) = 2300/3.24/900 ms, flip angle = 9°, matrix size = 256 × 256 × 224, 0.87 mm isotropic resolution, bandwidth = 220 Hz/pixel, and parallel imaging factor = 2. The ^1^H-MRS voxel was localized, as in [32], to the medial frontal cortex, encompassing part of the dorsal ACC and parts of the frontal pole using the MPRAGE images **(**Fig. 2**)**. ^1^H-MRS data were acquired using the MEGA-PRESS sequence [61] with the following parameters: voxel size = 20 × 30 × 30 mm^3^, TR/TE = 1500/80 ms, acquisition bandwidth = 1200 Hz; pulse placement edit-on/edit-off = 1.9/7.5 ppm, and number of excitations edit-on/edit-off = 96/96. Two MEGA-PRESS acquisitions were collected, interleaved with a water unsuppressed reference scan. The reference scan was acquired with the same parameters except water suppression pulses were not applied and number of excitations = 16. Due to co-edited macromolecule (MM) contamination, GABA+ (i.e., GABA with MM) was assessed.

### ^1^H-MRS data processing and quantification

^1^H-MRS processing was conducted in MATLAB (MathWorks Inc., Natick, MA) using Gannet [62]. Processing steps consisted of: 1. coil combination and prephasing based on the reference scan; 2. zero-filling of data to obtain nominal spectral resolution of 0.061 Hz/point; 3. eddy current correction [63]; 4. line broadening of 3 Hz; 5. frequency and phase correction using the spectral registration method [64] with correction parameters estimated from the edit-off data; 6. Difference spectrum estimation by pairwise subtraction of the edit-off data from the edit-on data. The two MEGA-PRESS acquisitions were processed independently and then averaged for a resulting difference spectrum generated from 192 edit-on and 192 edit-off excitations. The GABA (with MM) and Glx concentrations were then quantified from the average difference spectrum using TARQUIN [65] with the average processed water unsuppressed reference scan used to estimate concentrations relative to water.

In line with best practices, visual inspection of all spectra was used as quality control, as support for quantitative cutoffs such as the Crámer Rao Lower Bound (CRLB) as a basis for exclusion is equivocal and widely used thresholds may cause bias in estimated mean concentrations. Scans were excluded for excessive noise in the spectra or failure in model fitting for the GABA or Glx peaks. These exclusions were due to field inhomogeneity within the ^1^H-MRS voxel as a result of either poor shimming or participant motion, with the latter causing subtraction errors that result in model-fitting failure.

### Definition of variables used

- Pretreatment ${\rm{Glx}}/{\rm{GABA}}=\frac{{\rm{Pretreatment\; Glx}}}{{\rm{Pretreatment\; GABA}}}$. This ratio may better reflect biology than Glx or GABA alone, as these two neurotransmitters work in concert to regulate excitatory-inhibitory balance in the brain.
- *Days to response* were defined as the first time the participant obtained a HAM_17_ score <=50% of their pretreatment HAM_17_ score (regardless of whether they completed the 8-week trial).
- *Remitters* were defined as those who showed a 50% or more reduction from pretreatment HAM_17_ and with a posttreatment HAM_17_ of 7 or less at week 8.
- Percent difference in depression severity (note, the direction is defined such that positive values reflect improvement)=$\frac{({\rm{Pretreatment}}{{\rm{HAM}}}_{17}-{\rm{Posttreatment}}{{\rm{HAM}}}_{17})}{{\rm{Pretreatment}}{{\rm{HAM}}}_{17}}* 100 \%$

### Statistical analyses

Initial analyses considered possible differences between treatment and placebo cohorts. Chi-squared tests with exact *p*-values based on Monte Carlo simulation were used to examine the marginal association between categorical variables (*remitter, non-remitter*) and treatment type (*SSRI or placebo*). Wilcoxon rank sum tests were used to compare unadjusted marginal differences for any continuous variable (*age, pretreatment GABA, pretreatment Glx, pretreatment Glx/GABA, percent difference in depression severity, and days to response*) between treatment types. Spearman rank correlation coefficient was used to measure the linear relationship between outcome variables (*percent difference in depression severity and days to response*) and continuous variables (*pretreatment GABA, pretreatment Glx, and pretreatment Glx/GABA*) by *treatment type*.

Multivariable logistic regression models were utilized to determine the relationship between the outcome variable (*remitter vs non-remitter*) and pretreatment measure (*pretreatment GABA, pretreatment Glx, or pretreatment Glx/GABA*) after adjusting for *treatment type* and with/without *ag*e. Multiple linear regression models were further used to determine the relationships between outcome variables (*percent difference in depression severity and days to response*) and *pretreatment GABA or pretreatment Glx or pretreatment Glx/GABA* after controlling for *treatment type* (and with/without *age*). In all models, the treatment-specific relationship was first explored by including a two-way interaction term between *treatment type* and *pretreatment GABA or pretreatment Glx or pretreatment Glx/GABA*. As there was no strong evidence for significant two-way interaction terms, except for *treatment type with pretreatment Glx* having a significant association *with percent difference in depression severity*, other interaction terms were not included in the final regression models.

Bayes factor hypothesis testing [66] was further implemented for each regression model. A Bayes factor (BF) ≤ 1/10 indicates strong evidence for the null hypothesis (H_0)_, 1/10 < BF ≤ 1/3 indicates moderate evidence for H_0_, 3 ≤ BF < 10 showed moderate evidence for H_1_ (that a relationship exists between variables), BF ≥ 10 indicates strong evidence for H_1_, otherwise, 1/3 < BF < 3 indicates absence of evidence to support H_0_ or H_1_ [66]. The R package “BayesFactor” was used for linear regression and “BFpack” was used for logistic regression (Mulder et al., 2019) to calculate the Bayes factor. Bayes factor hypothesis testing was performed using R 3.6.1, and other statistical analysis was performed using SAS 9.4 and significance level was set at 0.05 (SAS Institute Inc., Cary, NC).

#### Treatment types

No significant difference was found for any continuous variables (*age, pretreatment GABA, pretreatment Glx, pretreatment Glx/GABA, treatment PD, and days to response)* between two *treatment types*. Even though placebo and SSRI may have different biological mechanisms, this result was in line with the past studies that found no difference in the response rates between placebo and treatment groups [67, 68]. This is further supported by reviews indicating less than half of remission from MDD is related to active treatment, with the remainder attributable to placebo effects and spontaneous improvement [69]. Treatment groups were, therefore, combined in all analyses to obtain optimal power, and treatment type was included as a covariate.

## Results

### Participant demographics

Demographics of participants included in this analysis are provided in Table 2. 6 participants’ imaging data were discarded due to poor quality (See ^1^H-MRS Data Processing and Quantification) and an additional 6 participants (*n* = 3 from the active treatment cohort and *n* = 3 from the placebo cohort) did not complete the trial, resulting in 73 participants for the primary analyses. In the exploratory study examining the days to response, 74 participants were included (note that one participant did not complete the trial but was in the study long enough for a response). Only responders (*n* = 51) were used for the study examining the days to response. For this analysis involving weekly/biweekly depression assessment, missing intermediate HAM_17_ scores during the 8-week period were not included in the analysis.

**Table 2:** Pretreatment demographics and clinical characteristics of study participants.

Characteristics	Remitters (*N* = 25)	Non-remitters (*N* = 48)	*p*-value^1^	Responders (*N* = 51)	Non-responders (*N* = 23)	*p*-value^1^
Age, years, mean (SD)	30.4 (15.7)	29.3 (12.7)	0.74	30.4 (14.5)	28.2 (11.5)	0.52
Range	18–64	18–65		18–65	19–60	
Sex, N (%)			0.43			0.28
Male	10 (40)	14 (29.2)		19 (37.3)	5 (21.7)	
Female	15 (60)	34 (70.8)		32 (62.7)	18 (78.3)	
Race/Ethnicity			0.13			0.72
Caucasian	15	30		33	13	
African American	2	2		2	2	
Asian	2	12		8	6	
Pacific Islander	1	0		1	0	
Mixed	5	4		7	2	
17-item Hamilton Depression Rating Scale, mean (SD)	15.9 (3.9)	18.8 (4.9)	**0.01**	17.5 (4.3)	18.3 (5.6)	0.50
Days of treatments, mean (SD)	58.7 (7.3)	59.5 (8.2)	0.46	59.0 (9.1)	58.5 (6.6)	0.28
Subtype of depression, N (%)			0.21			0.18
Atypical	6 (24)	4 (8.3)		9 (17.6)	2 (8.70)	
Melancholic	6 (24)	13 (27.1)		10 (19.6)	9 (39.1)	
No subtype	13 (52)	31 (64.6)		32 (62.7)	12 (52.2)	
DSM-IV comorbidity at time of study, N (%, *n*=number of unknowns)						
None	4 (17.4, unk = 2)	6 (13.0, unk = 2)		8 (16.3, unk = 2)	3 (14.3, unk = 2)	
OCD	0 (0)	2 (4.2)		1 (1.96)	1 (4.35)	
PTSD	2 (8.7, unk = 2)	13 (28.3, unk = 2)		10 (20.4, unk = 2)	5 (23.8, unk = 2)	
Alcohol Abuse	5 (20.0)	8 (16.7)		10 (19.6)	3 (13.0)	
Cannabis Abuse	6 (25.0, unk = 1)	6 (12.5)		8 (16.0, unk = 1)	4 (17.4)	
Any Other Anxiety Disorder	17 (73.9, unk = 2)	40 (83.3)		37 (75.5, unk = 2)	20 (87.0)	
Number of antidepressant trials, lifetime, N (%, n = unknowns)			0.50			0.69
None	15 (62.5, unk = 1)	24 (52.2, unk = 2)		26 (54.2, unk = 3)	13 (56.5)	
1	4 (16.7, unk1)	7 (15.2, unk = 2)		8 (16.7, unk = 3)	3 (13.00)	
≥2	5 (20.8, unk = 1)	15 (32.6, unk = 2)		14 (29.2, unk = 3)	7 (30.4)	

In parentheses are standard deviation (SD) or percentage of each variable, calculated as number of occurrence/totals with known data. ukn = number of participants for which this variable is not recorded, which is 0 unless specified.

^1^For categorical variables, *p*-value was based on a Chi-squared test with exact *p*-value from Monte Carlo simulation; for continuous variables, *p*-value was based on a t test.

Data from this study have been previously published; however, the previous studies involved either PET-only measures, changes in ^1^H-MRS with treatment [32, 60] or a combination of structural MRI and PET [70].

### Pretreatment GABA, Glx or Glx/GABA in remitters vs. non-remitters

**Fig. 3:**
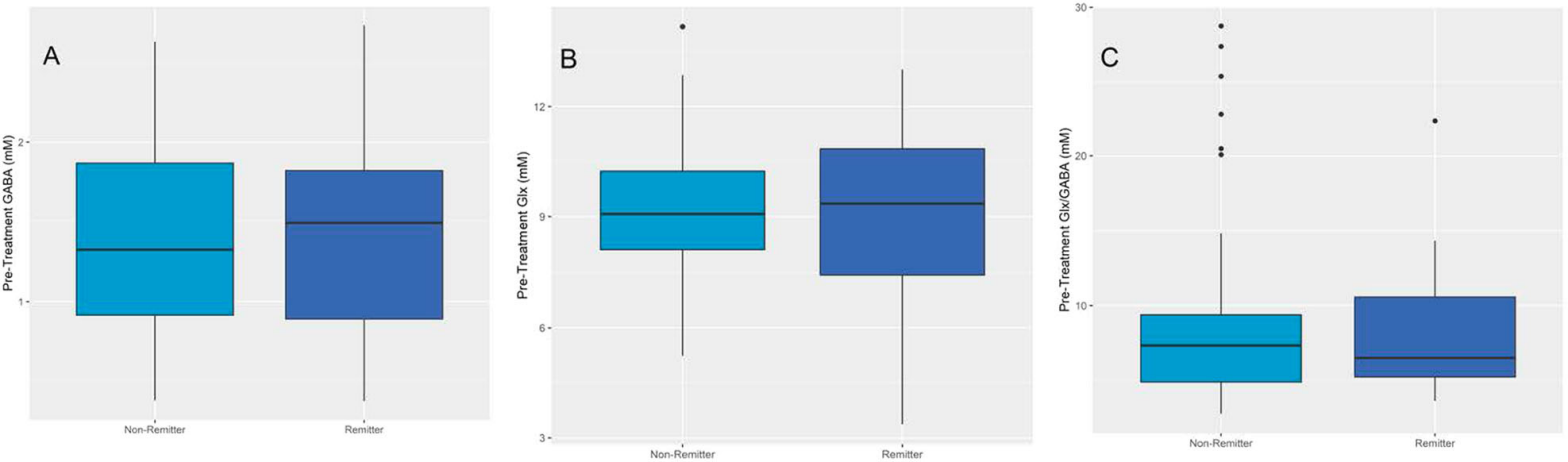
Pretreatment Metabolites Concentrations in Remitters and Non-remitters Group. Pretreatment GABA (**A**), Glx (a combination of glutamate and glutamine) (**B**) or Glx/GABA (**C**) in remitters and non-remitters. Error Bars are the 95% confidence interval. The bottom and the top of the box mark the 25 and 75th percentiles, and the line inside the box indicates the 50th percentile.

Fig. 3 shows pretreatment GABA, Glx, or Glx/GABA by remitter status. After adjusting for treatment type and age, there was no significant relationship between remitter status and pretreatment GABA (odds ratio [OR] = 1.06, 95% confidence interval [CI]: 0.48–2.34, *p*-value = 0.88), Glx (OR = 1.08, 95% CI: 0.83–1.40, *p*-value = 0.59) or Glx/GABA (OR = 0.97, 95% CI: 0.88–1.06, *p*-value = 0.47). Bayes factor analyses further reinforced these results by showing strong evidence for null hypothesis (BF = 0.01 in all cases). Conclusions were similar when age was removed as a covariate (BF for GABA = 0.03, Glx = 0.04 or Glx/GABA = 0.05).

### Relationships between pretreatment GABA, Glx or Glx/GABA and percent change in depression severity

**Fig. 4:**
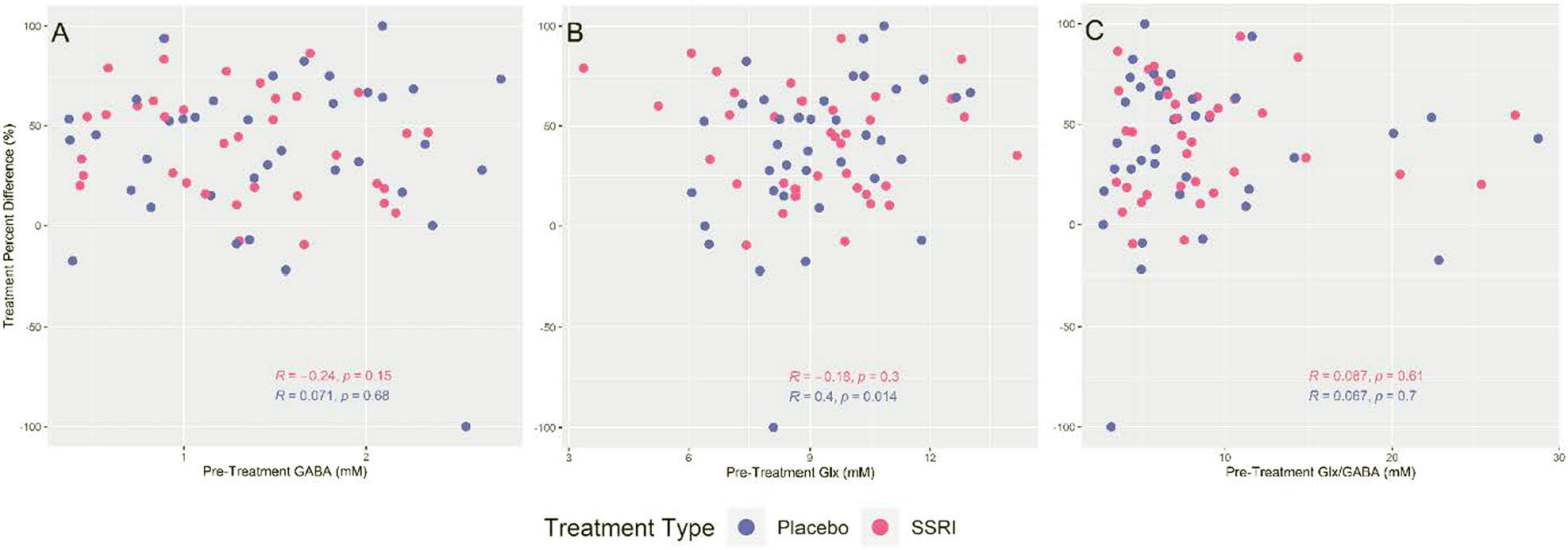
Correlation of Pretreatment Metabolites Concentrations and Percent Difference in Hamilton Depression Rating Scale. Percent difference in Hamilton Depression Rating Scale with treatment versus pretreatment GABA (**A**), Glx (a combination of glutamate and glutamine) (**B**) or Glx/GABA (**C**). Treatment type is indicated by color. Positive y-values indicate a reduction in symptoms. (*R*: correlation coefficient; *p*: *p*-value). SSRI selective serotonin reuptake inhibitor (escitalopram).

Fig. 4 shows the relationship between pretreatment GABA, Glx or Glx/GABA and percent decrease in depression severity by treatment type. After adjusting for treatment type and age, there was no significant relationship between percent decrease in depression severity and pretreatment GABA (estimated coefficient = −6.14, 95% CI: −18.89–6.61, *p*-value = 0.34), Glx (estimated coefficient = 1.81, 95% CI: −2.35–5.98, *p*-value = 0.39) or Glx/GABA (estimated coefficient = 0.12, 95% CI: −1.25–1.48, *p*-value = 0.87. Bayes factor analyses further reinforced this result by showing strong evidence for null hypothesis (BF for GABA, Glx = 0.04, or Glx/GABA = 0.03). Conclusions were similar when age was removed as a covariate (BF for GABA, Glx = 0.10, or Glx/GABA = 0.07).

The percent decrease in depression severity did show a significantly positive relationship with pretreatment Glx in the placebo group (estimated coefficient = 7.65, 95% CI: 1.40–13.90, *p*-value = 0.02). The result remains even after removing the outlier whose change in depression severity was −100%; however, this result would not survive multiple comparisons correction.

### Pretreatment GABA, Glx or Glx/GABA correlation with days to response in responders

**Fig. 5:**
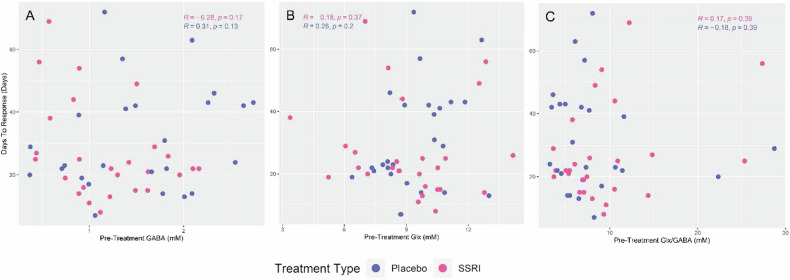
Correlation of Pretreatment Metabolites Concentrations and Days to Response. Days to response (in responders only) versus pretreatment GABA (**A**), Glx (a combination of glutamate and glutamine) (**B**) or Glx/GABA (**C**). Treatment type is indicated by color. (*R*: correlation coefficient; *p*: *p*-value). SSRI selective serotonin reuptake inhibitor (escitalopram).

Fig. 5 shows the relationship between pretreatment GABA, Glx or Glx/GABA and days to response in the responders by treatment type. After adjusting for treatment type and age, there was no significant relationship between days to response and pretreatment GABA (estimated coefficient = −0.82, 95% CI: −8.50–6.85, *p*-value = 0.83), Glx (estimated coefficient = 0.33, 95% CI: −1.91–2.57, *p*-value = 0.77) or Glx/GABA (estimated coefficient = 0.25, 95% CI: −0.54–1.05, *p*-value = 0.52). Bayes factor analyses further reinforced this result by showing strong evidence for null hypothesis (BF for GABA, Glx = 0.06, or Glx/GABA = 0.07). Conclusions were similar when age was removed as a covariate, with Bayes factor analyses showing moderate evidence for the null hypothesis (BF for GABA, Glx = 0.12, or Glx/GABA = 0.14).

## Discussion

The present study demonstrated that pretreatment GABA and Glx, at the current stage, are unlikely to serve as clinically useful biomarkers for antidepressant treatment response to SSRI or placebo treatment. Contrary to our hypotheses, we did not find that participants with lower pretreatment Glx levels were more likely to be remitters, and no significant correlation was found between pretreatment Glx level and participants’ treatment response. Though a significant relationship between pretreatment Glx and improvement in depression was observed within the placebo cohort only, this relationship is likely not clinically significant as only a small percent of the variance in change in depression severity is explained by pretreatment Glx (16%), the result would not withstand multiple comparisons correction, and the Bayes factor analyses indicated strong evidence for the null hypothesis.

The lack of a significant relationship between pretreatment Glx and outcome, across escitalopram and placebo cohorts, may have been a result of the Glx signal itself. It is hypothesized that the decreased glutamine observed in MDD is due to decreased conversion of glutamate to glutamine. Thus, successful treatment may require normalization of glutamate-glutamine cycling, increasing glutamine levels while reducing the excess glutamate levels at synaptic cleft. This may result in no net change in Glx, a combination of both signals, suggesting that it may be necessary to detect the individual levels of Glu and Gln to predict treatment response. In support of this hypothesis, a recent study conducted by Brennan et al, (2017) reported no change in Glx level in MDD after successful response to SSRIs [71]. Studies conducted to examine Glx changes in other treatment modalities, including rTMS, DBS, CBT, and ketamine, have also often yielded mixed results [72–76]. Additionally, our group reported no significant change in Glx with both SSRI and placebo in this cohort [32]. Therefore, a more comprehensive examination of Gln, Glu, Glx and even the Glu-Gln cycling using both ^1^H-MRS and ^13^C MRS may be required to predict outcome as well as assess treatment-induced differences [77]. This suggests that pretreatment Glx may not be predictive of treatment response to any active medication or placebo.

Similarly, model results did not show that pretreatment GABA levels were associated with remitter status to either SSRI or placebo, and no significant correlation was found between pretreatment GABA level and participants’ treatment responses. This result is in line with studies that have previously demonstrated no correlation between pretreatment GABA levels and the magnitude of subsequent clinical improvement with antidepressant treatments such as ketamine and SSRIs [10, 29]. Relatedly, our group has reported no differences in GABA levels with treatment in this cohort [32].

As we have previously described [32], the glutamate, glutamine, and GABA cycle through a shared synthetic pathway [78], therefore, the Glx to GABA ratio (Glx/GABA) may provide greater resolution than either neurotransmitter alone. Further, the ratio better reflects the biology, as these two neurotransmitters work in concert to regulate excitatory-inhibitory balance in the brain [79]. However, the present study also showed no relationship between this ratio and any of the clinical outcomes was found.

For completeness, we also performed all analyses in the SSRI cohort only (data not shown) and conclusions were unchanged. In addition, we examined whether there was an interaction between TRD status and model results (data not shown) because patients with TRD often have lower rates of response/remission to active treatment [80] and to placebo [81]. However, a review published recently has shown that both non-TRD and TRD MDD is associated with hyperactivity within the same regions, including the ACC [82], suggesting the neurobiological underpinnings may be similar. In the present study, no interaction was observed between TRD status and treatment type, and all original conclusions remained unchanged. This suggests that neither treatment type nor TRD status affected model results, i.e., the ability of pretreatment GABA or Glx to predict response.

In addition to examining the prediction of treatment response, an exploratory analysis was also included to examine the use of pretreatment GABA and Glx levels to predict time to treatment response. However, no significant correlation was found between the participants’ days to response and pretreatment GABA, Glx or Glx/GABA level. This result was not surprising given the lack of significant relationship with treatment response. Even though several candidate genetic variants for early response have been identified [83–85], no neurobiological signature of early response in MDD has been validated.

Two main limitations existed in this study. The first is that this MRS study, acquired at 3 T, was unable to distinguish Gln from Glu, due to the overlap of resonance frequencies for Gln and Glu. A 7 T ^1^H-MRS with higher B_0_ field is required to obtain quantitation of Gln and Glu levels separately. However, better peak resolution produced by 7 T is usually accompanied by increased spectral dispersion, which leads to a larger and more unacceptable chemical shift displacement error (CSDE) with only the standard pulse sequences such as Point Resolved Spectroscopy (PRESS) implemented [86–88]. Thus, instead of PRESS, the adiabatic selective refocusing (sLASER) sequence, with high bandwidth adiabatic full-passage pulses (AFP), should be utilized at 7 T ^1^H-MRS to generate high quality MRS data with clean localization and minimal CSDE [89]. In the absence of these techniques, a combination of glutamate and glutamine (Glx) was observed in this study instead.

The second limitation resides in the heterogeneity of MDD. Utilizing a stratified design based upon various aspects of MDD may be a reliable way to address this heterogeneity [90, 91], for example, incorporating clinical characteristics, genetics and neuroimaging [92]. Our recent study also reinforced the necessity of integrated information by discovering that 2-[^18^F]-fluorodeoxyglucose-Positron Emission Tomography (FDG-PET) signal alone was not predictive of SSRI treatment response in MDD [60]. Along with the inability of MRS alone to be predictive of response found in this study; these results suggest that a combination of imaging techniques may also be needed to fully distinguish MDD treatment response.

Even though limitations did exist, several advantages should be noted. The study involved a large cohort of participants (*N* > 70). All participants were either washed out of medication or medication-free at least three weeks prior to imaging, preventing effects of previous medications from confounding the pretreatment assessments. Treatments were administered systematically, in a double-blind placebo-controlled trial, and ratings of depression severity were performed by experienced raters. While the inclusion criteria were assessed with the MADRS, this was separate from the primary outcome measure (HAM_17_) to prevent potential artificial inflation of pretreatment depression score. Further, all the analyses were computed by rigorous statistical analyses, including Bayes Factor analysis which provides evidence for the strength of support for the null hypotheses. Additionally, sex and age were included as covariates since there are sex differences in response to SSRI in MDD [93].

## Conclusion

In conclusion, this study is the first to examine GABA, Glx and Glx/GABA as potential treatment response biomarkers for MDD using a large sample size and with a placebo-controlled cohort. A significant relationship was not found between pretreatment GABA, Glx or Glx/GABA and remission status, change in depression severity with treatment, or days to response. This finding is not unexpected given the equivocal results found in previous studies (Table 1) and suggests that resources should be diverted from similar studies to alternate approaches including multimodal approaches, an important consideration for future study design.

## Data Availability

The data that support the findings of this study are available from the senior author, CD, upon request.
